# Extracellular vesicles as new-age vaccine carriers: a focused account on viral diseases

**DOI:** 10.3389/fimmu.2026.1820746

**Published:** 2026-06-19

**Authors:** Roopali Rajput, Madhu Khanna, Radhika, Jitender Sharma, Hardeep Kaur

**Affiliations:** 1 Virology Unit, Department of Microbiology, Vallabhbhai Patel Chest Institute, University of Delhi, Delhi, India; 2 Department of Zoology, Ramjas College, University of Delhi, Delhi, India; 3 Department of Biochemistry, Govind Ballabh Pant Institute of Postgraduate Medical Education and Research, New Delhi, India

**Keywords:** drug delivery, EV engineering, exosome, vaccine delivery, vesicle, viral vaccine

## Abstract

Extracellular vesicles are shifting the paradigm in vaccine development by virtue of their diverse benefits as carriers of immunogens to targeted hosts. These nano-sized naturally occurring biomolecules offer safety, transmissibility across membranes, excellent presentation of antigens and robust immune responses when engineered as vaccine delivery vehicles. Given the remarkable potential of these nanoscale biological carriers, the scientific community has witnessed substantial escalation in research activity highlighting their applications in vaccines and drug delivery. However, the research is still in its early stage and the scientists are actively striving to optimize the engineering of these molecules for effective therapeutic applications. As a research group working on similar lines, we sought to explore the diverse engineering strategies with the aim of understanding the potential of extracellular vesicles as potent vaccine delivery vehicles. We conducted a systematic search to critically screen original research studies investigating engineering of extracellular vesicles for production of vaccines against viral infections. Our analysis of the studies revealed that extracellular vesicles provide diverse engineering opportunities to enable efficient viral antigen presentation, and to elicit robust immune responses both *in vitro* and *in vivo*. A wide spectrum of viruses has been targeted driving development of versatile as well as pathogen-specific vaccine strategies. Advancing research in this domain could deepen mechanistic insight into viral infections, while opening novel pathways for translational application, drug delivery and vaccine design. We conclude that substantial opportunities remain to fully harness the potential of extracellular vesicles in biomedical research, particularly in context of viral infections.

## Introduction

Extracellular vesicles (EVs) are defined as particles released from cells having a lipid bilayer and unable to replicate (1). EVs are naturally released by all organisms ranging from prokaryotes to multicellular eukaryotes. EVs can be derived from cell culture and engineered to present specific proteins or deliver desired cargo to designated targets. These particles are believed to be essential means of transporting different biological molecules, like, sub-types of RNA, metabolites, peptides, lipids that are selectively loaded into the (2). Humans secrete EVs from all cell types and into all body fluids (2). Lately, EVs have become the vehicle of choice for drug and vaccine delivery owing to some of the key features, such as, high biocompatibility due to natural origin, protection of cargo from enzymatic degradation in body fluids by virtue of lipid bilayer, easy transfer across blood-brain barrier (BBB) due to their structure (3). The robust potential of EVs is evident by the progressive increase in the number of research studies and publications on their applications in vaccinology or drug development and delivery. Nevertheless, this key area of biomedical research is still blossoming and there is immense scope of exploration of strategies to utilize the potential of EVs in targeted delivery of drugs and vaccine candidates. Once established, any potent strategy can be robustly applied to cater to most of the prevailing human diseases of concern, especially infectious diseases, as these require urgent attention across the globe.

For instance, EVs that were extracted from respiratory syncytial virus (RSV)-infected cells demonstrated higher level of innate immune activation as evident by significant release of cytokines and chemokines, as compared to EVs from uninfected control cells (4). RSV-infected cell derived EVs, when administered to A549 cells, downregulated RSV replication. Likewise, another study on the Foot and Mouth Disease Virus (FMDV), demonstrated the potential of EVs expressed from FMDV infected antigen presenting cells (APCs) in inducing virus-specific immunity. This was observed by virtue of FMDV cellular and antibody response-stimulating antigens in the virus infected APC EVs (5). The study further reported that the EVs showed presence of CD9, CD81, CD63 (conventional markers for EVs) and CD86 and MHC class II (immune-modulatory markers). Development of vaccines to counteract specific flavivirus infections has encountered various challenges. However, flavivirus-specific circulating non-structural 1 (NS1) protein remains a target-of-choice for Dengue virus (DENV) or Zika virus (ZIKV) vaccines (6, 7). EVs carrying DENV- or ZIKV-NS1 dimers demonstrate vaccine potential (8); however, complex immunopathogenesis remains a major hurdle (9). ZIKV infected human brain microvascular endothelial cells were shown to generate EVs harboring viral RNA, NS1 and Envelope (E) proteins, capable of transferring the viral components across BBB to the other cells of central nervous system (10). This demonstrated the potential of EVs as ZIKV vaccine carriers. Similarly, exosomes emerged as one of the key strategic targets to optimize MRC-5 cell-based rabies vaccine (11).

Given the pivotal roles of EVs in human viral infections, EV-engineering strategies facilitating their use as viral vaccine delivery agents were studied. A search on PubMed using the terms, “extracellular vesicles”, “vaccine”, and “virus” generated 247 results till December 15, 2025. Only ‘full text research articles published in English language’ were screened for inclusion. Of the initially screened 109 articles, 38 relevant studies were finally selected. A major portion of the selected articles displayed strategies for anti-SARS-CoV-2 vaccines followed by viruses- influenza, Zika, Coxsackie, Japanese encephalitis, etc.

## EVs: biological foundations and engineering opportunities

### Definition of EVs

According to MISEV2023, the term “extracellular vesicles” may be defined as “particles that are released from cells, are delimited by a lipid bilayer, and cannot replicate on their own” (1). By this definition, it is imposed that EVs possess no functional nucleus, meaning that these nanoscale entities cannot multiply in numbers on their own.

### Classification of EVs

EVs may be classified according their biogenesis (exosomes, ectosomes or apoptotic bodies), to physical properties (mimetics, synthetic, artificial cell-derived), or particle size (small EVs, large EVs). EVs are termed ‘exosomes’ when the nanoparticles originate via multivesicular body (MVB) from internal cellular compartments. Ectosomes are EVs that arise from cellular surface. Cell processes, like, migration produces EVs that are termed as ‘migrasomes’, and programmed cell death generates EVs that are known as apoptotic bodies. It is worth to note that production of EVs can be natural (routine bodily functions) or stimulated (*in vitro* to enable research studies). Presently, there are no established biogenesis-based EV characterization methods. EV suspensions obtained either from clinical specimens (e.g., body fluids or tissues), or experimental sources (eg., animals or cultured cells) cannot be selectively enriched for specific EV subtypes; instead, these encompass the full spectrum of EV diversity (1).

### Sources of EVs

EVs are generated from a range of cell types, prokaryotes, eukaryotes, cell culture, plants, animals, etc. Human body fluids, such as, blood, urine, saliva, cerebrospinal fluid, synovial fluid, milk all are known producers of extracellular vesicles (1). Both Gram-negative and Gram-positive bacteria produce EVs. The nanoscale particles from Gram-negative bacteria are called as Outer Membrane Vesicles (OMVs), which chiefly bleb/lyse through bacterial outer membrane speculatively comprising periplasmic and inner membrane contents. OMVs that have been engineered to be used as viral vaccine carriers are reviewed here in subsequent sections. Blood followed by urine are the most investigated EV sources among the various human body fluids (1). For vaccine studies, most of the engineering strategies derived EVs from cultured mammalian cells or bacteria.

### Characteristics of EVs

EVs are known to contain a range of biomolecules, such as, lipids, nucleic acids and proteins. The outer layer of EVs is made up of phospholipid bilayer serving dual purposes of securing EV contents and transport vehicle (12). Phosphatidylserine, cholesterol, sphingolipids and ceramides comprise the lipid components of EVs. Nucleic acids, such as, dsDNA, ssDNA, mtDNA, mRNA, miRNA, small nuclear RNA, non-coding RNA, and small cytoplasmic RNA, have been also found in EVs. Further, EVs contain proteins, such as, enzymes, signal proteins, cytoskeletal proteins, chaperones and multivesicular bodies, either adherent to phospholipid bilayer or being carried internally. The repertoire of biomolecules associated with EVs is steadily expanding (13).

## EV-based vaccine platforms: engineering strategies

### Universal vaccine approaches

**Figure 1:**
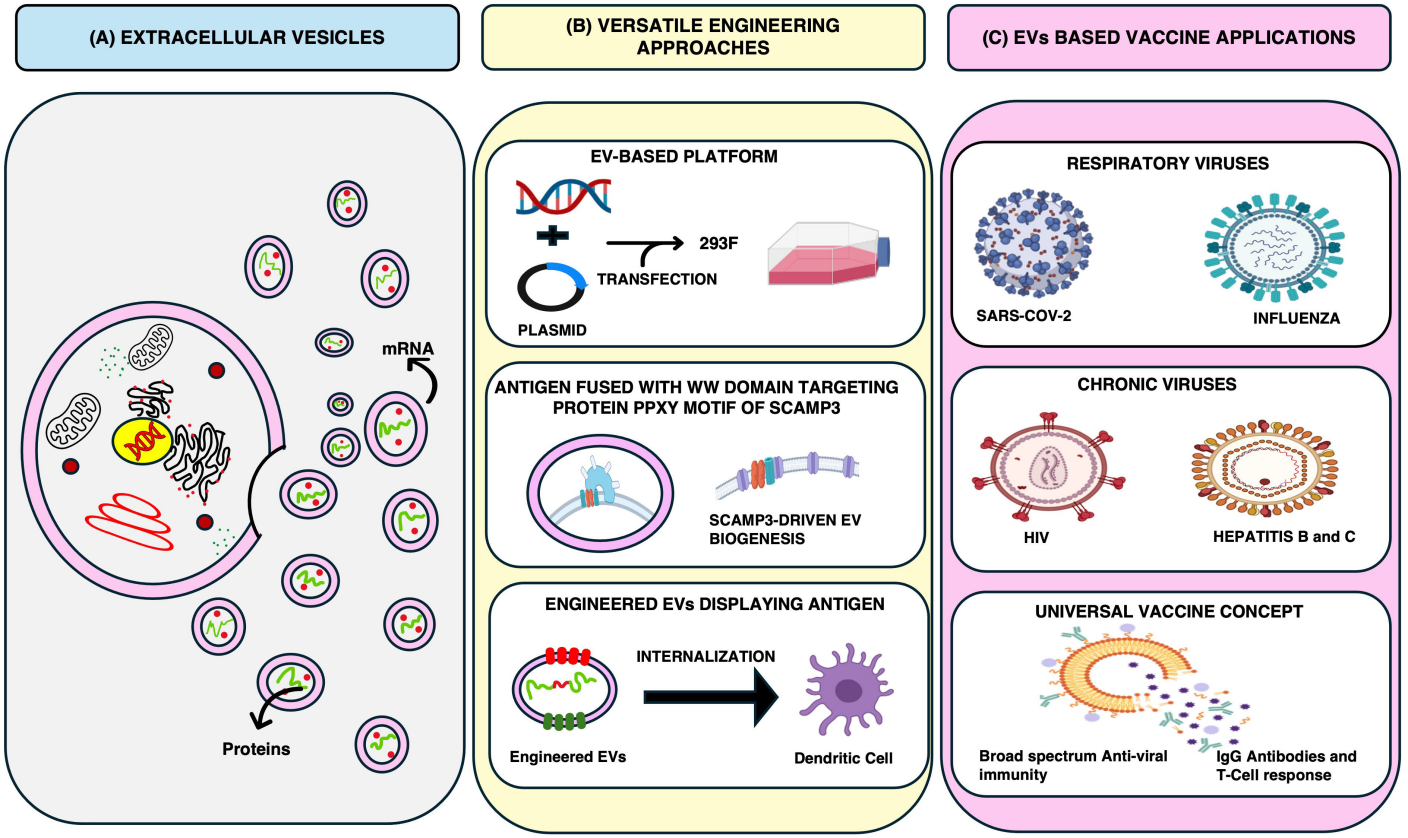
Engineering approaches for Extracellular vesicles (EV) based vaccine platforms aiming to combat infectious diseases.

EVs have served as carriers for transporting different types of cargo to specific targeted organelle, tissues or cells, enabling diverse targeted engineering opportunities to develop cutting-edge vaccine platforms (**Figure 1**) for a range of human diseases (14, 15). One of the recent studies explored the potential of EVs as a template for developing explicit vaccine for any infectious disease or cancer (16). The researchers termed their vaccine strategy as the EV^X-M+P^ platform, wherein any targeted gene cloned in lentivirus vector and expressed in 293F cells can facilitate delivery of mRNA and protein as vaccine candidates, thereby inducing humoral as well as adaptive immunity (**Table 1**). The authors validated the platform against melanoma tumors by expressing ovalbumin (EV^OvaM+P^) and SARS-CoV-2 by expressing Spike (S) protein (EV^SpikeM+P^). Robust protective neutralizing IgG antibodies and T cell responses in mice or baboons were recorded (16).

**Table 1:** Universal vaccine platforms based on engineered extracellular vesicles.

Reference	Virus targeted	Vaccine candidate	EV engineering	Host	Immunity achieved	Advantages	Disadvantages	Scope of application
Universal vaccine strategies								
(16)	SARS-CoV-2	mRNA + protein	• EVX-M+P vaccine	• Mice • Baboons	Humoral and adaptive responses	• Both protein and mRNA transported as vaccine candidates,generating more effective immunity. • Lesser quantity of antigen is required.	• Cost-exhaustive initial set- up • Technical precision required.	Universal
(18)	Influenza virus & HIV	Peptide or Proteins	• WAEV (WW domain activated EVs)	• HEK293T cells • Mice (CD-1 IGS and BALB/ c)	Humoral responses; Protection from lethal influenza virus challenge	• Definite surface display of antigens in native form. • Not impacted by pre- existing or stimulated neutralizing antibodies. • Generally stable. • Cost-effective storage, and logistics. • Optional usage of adjuvant.	• Technical expertise and sophisticated handling required. • Specific host- testing required before results.	Universal
(19)	SARS-CoV-2	Protein	• SARS-CoV-2 S Δ21 carrier of vaccine epitope • VSV G fusion of S1 carriers of vaccine epitope • Pre-requisites- target antigen fused with SARS-CoV-2 S2 subunit (acting as a carrier to transport target antigen for immune stimulation)	• Renca cells	Humoral responses	• Rapid and cheaper production of unlimited amount of native form of target antigen. • No non- specific immune activation (no freely circulating antigen). • Other viral proteins analogous to SARS-CoV-2 S protein, such as, influenza HA, RSV F glycoprotein, and HIV gp160 (env) may be used as carriers. • No need of adjuvant	• Compatibility of target epitope and carrier protein need to be determined. • Technical expertise required for designing the framework.	Wide- Viral, bacterial diseases, & cancer.
(20)	Targeted approach for enveloped viruses/VLPs	Protein	Co-display of two GPI-anchored proteins on OMV surface	NA (Only display strategy demonstrated)	NA	• External adjuvant supplementation not required. • Simultaneous display of heterologous proteins	• Purification and hence toxicity concerns of the engineered OMVs	Wide- Any enveloped virus or VLP
Pathogen-specific vaccine strategies								
Reference	Virus targeted	Vaccine candidate	EV engineering	• Host	Immunity achieved	Advantages	Disadvantages	Scope of application
(23)	SARS-CoV-2	Protein	Virus-mimetic nanovesicles (VNV)	• Mice (BALB/c)	• Humoral immunity • IFN-γ and IL-2 cytokines	• VNVs exhibited higher yield than EVs • Homogenous surface display of antigen • Functional antigen even post-chemical treatment	• Adjuvant required	Other human diseases
(24)	SARS-CoV-2	Chemically synthesized linear B-cell S protein epitopes having a modified lysine containing an azide group at the N-terminus	Chemical conjugation of viral epitope on EVs	*In vitro* antibody recognition using COVID-19 patient sera	Humoral responses analyzed in patient sera	• Extremely time-efficient • Can expedite early vaccine development stages • Toxic copper-free • Azide group containing any molecule	• Interdisciplinary approach- Effective collaboration of teams essential • Pure formulations to be obtained by ultracentrifugation or ultrafiltration methods • Essential presence of azide group in target epitope • Cell culture or in vivo validation missing	Versatile
(25)	SARS-CoV-2 including delta variant	Recombinant Protein	• RBD‐conjugated outer membrane vesicles	• Golden Syrian hamster	RBD-specific antibodies Humoral Immunity	• Systemic and local responses • Esay administration	• Expensive • Concerns related to post translational modifications, such as, glycosylation of the target recombinant protein.	Versatile
(26)	SARS-CoV-2	Multi-subunit	sEVs containing both sRBD and Nucleocapsid antigens	• Mice	RBD-domain and nucleocapsid specific antibodies Humoral Immunity	• Robust Immune response • Personalised immunotherapy	• Production Challenges • Antibody escape mutants	Versatile
(27)	SARS-CoV-2	mRNA	m-RNA containing oEVs from Citrus sinensis	• Rats	Antigen-specific IgM, IgG and IgA santibodies	• Stable at room temperature • Optimal mucosal absorption	• Formulation was not fully optimized to evaluate raw effect of EVs on vaccination	Large scale vaccination
(28)	SARS-CoV-2	Protein	Genetically engineered dendritic cells in altered extracellular blebs (EBs)	• C57BL/6 Mice	Neutralizing antibodies against S-pseudotype	• Stability • Tolerate lyophilization	• Proof of concept strategy	Useful in locations with limited cold chain transportation storage facilities
(29)	SARS-CoV-2	DNA	Viral S1 or N proteins expressed as fusions with Nefmut	• C57BL/6 Mice	Protein S1 and N-specific CD8+ T cell	Potent CD8+ T cell response	• Immunity waning	Respiratory Viruses
(30)	SARS-CoV-2	Nucleocapsid	N-specific engineered EVs	• K18-hACE2 mice	N-specific CD8+ T cell	Active protection persisted 3 months post-immunization Potent with low mutation rate of antigen	• No dose-response curve • Lacks data on the specific traits of CD8+ T cells	SARS-CoV-2 and Variants
(31)	SARS-CoV-2	Protein	Spike protein- fused to the G protein of vesicular stomatitis virus EVs	• Human PBMCs	T-cell immunity	Effective immunity	• High donor variability	Versatile
(32)	SARS-CoV-2	Conjugated Protein (Inhaled form)	Conjugating RBD onto lung surfactant derived exosomes (RBD-Exo)	• Mucosal surface	Neutralizing antibodies, mucosal secretory IgA and T-cell response	Enhance local mucosal immunity. Reduce viral entry into lung epithelium	• Require storage temperatures (−20 °C or −70 °C) • High transit cost	Versatile
(33)	SARS-CoV-2	Conjugated Protein	Human lung spheroid cells derived exosomes (LSC-Exo)	• Mice	Neutralizing antibodies	Potent immunity	• Batch to batch variability	SARS-CoV-2 and Variants
(34)	SARS-CoV-2 delta variant	Protein	Spike protein via exosomes (STX-S)	• BALB/c mice	Neutralizing antibodies, CD8+ T cell and CD4+ T cell response	• Specific immunity against delta variant • Cross-immunity against Omicron variant BA.1 and BA.5 • Highly stable	• Lack of direct live viral challenge • Batch inconsistency	Versatile
(38)	Influenza	Adjuvant for protein	OMV with attenuated endotoxicity (fmOMV) by modifying the structure of the lipid A moiety of LPS	• C57BL/6 mice	Humoral and cellular immunity	• Attenuated endotoxicity • Systemic and mucosal immunity	• Expensive purification • Reduced potency	Intranasal vaccine adjuvant against Influenza
(39)	Influenza	Mucosal adjuvants for recombinant protein	Bone marrow derived dendritic cells (mDC-EVs)	• Balb/c mice	Humoral and cellular immunity	• Humoral and cellular immunity in blood and mucosa • Broader cross-protection against homologous and heterologous flu strains	• Require ultra-low temperature storage • Short shelf life • Scalability issue	Adjuvant for the development of mucosal vaccines against Influenza
(40)	Influenza A (H1NI & H3N2) viruses	Protein	rOMV based subunit	• BALB/c, C57BL/6 and DBA/2J mice • Ferrets	Humoral immunity	• Safety and potency • Robust immunity	• Rodent data less predictive of human dosing • Complex purification and low yields	Broad protection against Influenza strains
(46)	CVB3	Protein	Exo-VP1	• Mice	Humoral and cellular immunity	• High efficacy • Balanced immune response • High bioavailability	• Storage and stability issues • High production cost	Versatile
(48)	ZIKV	Protein	EVs-IFITM3	• Pregnant Mice	Intrinsic innate immunity	• Crosses placenta with high safety • Targeted mechanism with high efficacy • Biocompatible vehicle	• Short half life • Invasive delivery • Unknown long-term safety	Versatile
(49)	JEV	Recombinant DNA-based live-viral vectors	ALVAC vector and subunit EV	• Mice	Humoral immunity	• High biosafety • Large antigen insertion • Bypass pre-existing immunity	• Limited single dose efficacy • High cost	Versatile
(50)	RSV	Oligopeptides	RSV-specific peptide engineered DC exosomes	• C57BL/6 Mice	Cellular immunity	• Intact EV recovery • High specificity • No genetic modification of parent cells	• Complex multistep synthesis • Poor in vivo priming	Versatile
(51)	DENV	Protein	Δ agrMV bacterial vesicle platform	• Mice-adult and sucking	Humoral immunity	• Multi-antigen delivery • Reduced risk of antibody dependent enhancement • Safety	• Risk of anti-vector immunity • Residual endotoxins	Versatile
(52)	HPV	Protein	HPV-E7 fused to modified HIV-1 Nefmut	• Mice	Cellular immunity (CTLs)	• Highly potent CTL response • High density cargo packing • No anti-vector immunity	• Low yields • Complex purification and heterogeneity	Broad
(53)	HIV-1	Protein	Ova-Texo and Gag-Texo (T-cell based exosome arming)	• C57BL/6	Cellular immunity (CD8+ T cell)	• Dual delivery-antigen and CD40L • Low systemic toxicity • Reverses CTL exhaustion	• Potential for auto-immunity • Risk of viral vectors • Requirement of patient specific donor cells	Broad
(54)	PRRSV	Protein	EV-enriched fractions from convalescence animals	• Pig	Humoral and cellular immunity	• High safety • Differentiate between infected and vaccinated animals • Potent	• Variable humoral response • High cost	Limited
(55)	PRRSV	RNA	Sn- and CD163-targeted amiRNA vesicles	• Pig	Intracellular innate-like resistance via gene silencing (RNA interference)	• Strain independent resistance • No risk of viral reversion • Broad cellular distribution	• Transient protection • Complex production and high cost • Potential off-target effects	Versatile
(56)	IBV	ssRNA	HD11M1-exo from lipopolysaccharide (LPS)-activated HD11 macrophages	• Chicken	Humoral and cellular immunity	• Mucosal immunity • Biocompatible and safe	• LPS contamination risk • Storage and stability issues	Broad
(57)	Pseudorabies virus	Recombinant protein	Bacterial biomimetic vesicles (Low‐endotoxin *Salmonella choleraesuis* strain SC‐L3 with lipid A modification)	• Mice	Humoral and cellular immunity	• Mucosal immunity • Oral delivery • Safety and versatility	• Complex manufacturing • Cold chain dependence • Incomplete detoxification	Versatile

Most of the membrane viral proteins exhibit conformational epitopes, preserving which is difficult in an *in vitro* vaccine candidate. In a very interesting study, this concern was addressed by presenting viral antigens onto the surface of EVs by virtue of a modular interactable protein domain, the WW domain. WW domains are protein modules that enable protein–protein interactions by binding proline-rich motifs and phosphorylated Ser/Thr-Pro sites (17). In a serendipity experiment, engineered EVs were generated through interaction of WW domains with secretory carrier-associated membrane protein 3 (SCAMP3). EVs displaying influenza matrix 2 (M2) protein and hemagglutinin stalk (HA2 subunit), along with the conserved HIV gp41 MPER peptide, elicited significant antigen-specific antibodies. This comprises a universal vaccine approach (**Table 1**) as any targeted antigen can be displayed on the EV surface by fusing the desired foreign protein to the WW-domain that would in turn interact with the SCAMP3 and cause the budding of engineered EVs presenting the foreign protein/peptide over the EV surface (18).

Another meticulously planned versatile vaccine strategy (**Table 1**) utilizing exosome mimetics demonstrated efficacy in eliciting immunity against SARS-CoV-2 (19). Based on careful investigation of packaging and signaling leading to ectopic expression of the viral S protein, the researchers deleted a 21-amino acid intracellular signal sequence present at the protein’s cytoplasmic tail (S Δ21 protein). The deletion intensified S protein expression on plasma membrane causing budding of exosome mimics displaying the viral vaccine candidate leading to more efficient and safe immune responses compared to mRNA or inactivated virus vaccines. Further, fusion of the G transmembrane domain of the Vesicular Stomatitis Virus with the S1 subunit of the SARS-CoV-2 showed efficient membrane localization, analogous to the S Δ21 protein. The approach can utilize other transmembrane protein subunits transporting diverse antigens for developing safe and robust vaccines against any targeted pathogen (19).

An interesting molecular painting approach (**Table 1**) was employed to functionally modify the surface of *E. coli* OMVs with glycosylphosphatidylinositol (GPI)-anchored protein, as an attempt to present/co-present perfectly folded eukaryotic proteins on prokaryotic platform (20). This versatile approach can be used to develop vaccines against enveloped viruses, such as, influenza viruses, lentiviruses, coronaviruses, or herpes viruses, owing to the viruses’ intersecting biophysical attributes with EVs or OMVs.

### EV-based COVID-19 vaccines

Recent COVID-19 pandemic considerably paused the world in various ways. A recent analysis of more than 12,000 mammalian virus-host interactions offered an interesting reflection. The study reported that enveloped viruses are predisposed to causing cross-species zoonotic infections than viruses without an envelope (21, 22). With an intent to circumvent unprecedented future epidemics or pandemics, an innovative virus-mimetic nanovesicle (VNV) based vaccine strategy was devised (23). VNVs displaying the SARS-CoV-2 S protein elicited robust systemic and mucosal antigen-specific antibody responses, along with cytokine induction, in BALB/c mice (23). VNVs demonstrated higher yield and homogenous display of antigen, culminating in better S-specific immune responses than EVs. In another cutting-edge chemical conjugation approach, high density SARS-CoV-2 S protein display was achieved in just a few days, employing the strain-promoted azide−alkyne cycloaddition (SPAAC) reaction (24). This advanced click chemistry approach offers novel insights into.

The biological functionalization of COVID-19 vaccines encounters concerns, such as, maintenance/shelf-life issues, booster doses, desirable route of administration, and need to modify the principal immunogen as per emergence of variants. Nevertheless, most of the SARS-CoV-2 vaccines employ S, Nucleocapsid (N), or Envelope (E) as viral antigens of choice. *Salmonella typhimurium* EVs expressing cellularly produced receptor-binding domain (RBD) of S protein of SARS-CoV-2 were generated and characterized to address the above challenges (25). Golden Syrian hamster immunized with engineered EVs produced systemic and local RBD-specific antibodies against the wild-type and Delta variants (25). Another EV-based multi-subunit vaccine harboring spike protein RBD-domain and nucleocapsid epitope generated prolonged robust immune responses against SARS-CoV-2 infection in mice (26). An oral mRNA vaccine encoding the SARS-CoV-2 S1 protein subunit was developed using EVs from edible orange (*Citrus sinensis*) juice, and triggered antigen-specific IgM, IgG, IgA antibody responses in rats. The lyophilized and encapsulated EVs remained stable at room temperature for one year demonstrating a promising, stable oral vaccine platform (27). Another innovative strategy chemically modified genetically engineered dendritic cells expressing the SARS-CoV-2 spike protein into extracellular blebs (EBs). These altered EBs demonstrated the potential to elicit neutralizing antibodies against S-pseudotyped virus in C57BL/6 mice, highlighting the strategy as a safe vaccine approach (28).

Humoral responses are required for rapid clearance and neutralization of viral antigens; however, strong CD8+ T cells are necessary to circumvent the frequent concerns of ineffective antigen-antibody reactions due to frequent mutations in the viral antigens. EVs engineered to express S1 subunit of Spike protein (S1) or N of SARS-CoV-2 as fusions with an EV-anchoring protein, Nef^mut^, were intramuscularly delivered to experimental C57BL/6 mice via pVAX1 DNA vector. Potent S1- and N-specific CD8+ T cell responses were generated, while protection from lethal challenge was observed from elevated levels of N-specific CD8+ T lymphocytes (29). Another study by the same research group demonstrated active protection from experimental SARS-CoV-2 infection in K18-hACE2 mice by robust N-specific CD8+ T cells that were generated by immunization with N-engineered EVs. The level of protection persisted after 3 months of immunization by the N-specific resident memory cells, highlighting the potency of the choice of the viral antigen which has low mutation rate, rendering N-specific EVs possibly effective against emerging viral variants (30). Spike protein-specific EVs targeted to human peripheral blood mononuclear cells (PBMCs) generated effective T cell immunity (31). Inhalable COVID-19 vaccine was also developed by conjugating the RBD onto lung surfactant-derived exosomes (RBD-Exo), forming virus-like particles. Unlike intramuscular vaccines, inhaled RBD-Exo stimulated neutralizing antibodies, mucosal secretory IgA, and lung T-cell responses, effectively enhancing local immunity and reducing viral entry into the lung epithelium (32). Further work by the same research group demonstrated potent neutralization of SARS-CoV-2 and its variants and mice protection by engineered human lung spheroid cells (LSC)-derived exosomes (LSC-Exo) expressing the Angiotensin-converting enzyme 2 (ACE2) (33).

Another study utilizing a proprietary exosome-engineering platform, the (Capricor’s StealthX™) demonstrated delivery of Spike protein of the SARS-CoV-2 Delta variant (STX-S) via exosomes. The exosome-based vaccine triggered robust antigen-specific (Delta variant) and cross-protective (Omicron variants: BA.1 & BA.5) neutralizing antibody responses, as well as significantly augmented CD4+ and CD8+ T cell responses. This strategy demonstrated expanded immunological coverage than the existing COIVD-19 mRNA vaccines (34). Investigation of the kinetics of the COVID-19 vaccines employing the Spike protein as the main immunogen, revealed that circulating exosomes with the viral antigen induced humoral and cellular immunity that increased post-booster, and declined after four months (35).

### EV based Influenza virus vaccines

Respiratory viruses, such as, influenza continue to impose severe threat worldwide despite progress in novel antiviral candidates, diagnostic approaches, or surveillance strategies (36, 37). At present only live-attenuated vaccines are available for boosting mucosal immune responses. In view of providing an effective intranasal adjuvant to influenza vaccines, low endotoxic outer membrane vesicles (OMVs) derived from *Escherichia coli* were engineered to express a modified lipopolysaccharide (LPS), significantly enhancing humoral and cellular immunity in C57BL/6 mice (38). Another study with similar goal utilized EVs from mature bone marrow-derived dendritic cells (mDC-EVs). *In vitro* and *in vivo* analysis demonstrated excellent stimulation of APCs, and heightened humoral and cellular immunity in mucosa as well as blood of Balb/c mice (39). Distinct recombinant OMV (rOMV) based vaccine strategy explored the potential of lipid IVa portion of LPS in eliciting anti-influenza immunity in animal models (40). OMVs derived from these remodeled lipid formulations expressed the matrix 2 (M2) protein on the former’s surface and conferred complete protection upon lethal influenza A H1N1 and H3N2 virus challenge in BALB/c, C57BL/6, and DBA/2J mice. Also, considerably decreased viral titers in lungs of human pandemic H1N1 virus infected ferrets, demonstrating safety and potency of a relatively simple rOMV-based subunit vaccine modality (40).

Influenza viruses are notorious pathogens, known for frequent mutations especially in surface proteins, rendering ineffective immune responses from existing vaccines. Robust T cell immunity comes to rescue in such case, and with advances in EV-based vaccines, curiosity arises whether cross-presentation of an antigen to the professional APCs is possible. This information is critical as the advent of EV-based vaccines eliciting effective T-cell responses, by activation of naïve T-cells, may require presentation of epitopes that are not directly acquired by APCs (41). Furthermore, such immunogen transfer (cross-presentation) also enables evading influenza virus-induced inhibition of the host immunity (42). A classical study highlighted the role of exosomes, along with viral antigen’s specific domains, in potently transferring an HA epitope via chaperone H-2M to antigen presenting cells. Also, the receptor binding activity of HA improved exosome-mediated transfer of another surface protein, demonstrating a unique and possibly universal vaccine strategy to obtain elevated CD4+ T cell responses (43).

### EV-vaccines against other human viral infections

EVs are becoming a mode of choice for developing vaccines against some of the major human viral infections worldwide. Coxsackievirus B3 (CVB3) is a major etiological agent of viral myocarditis (44). Lack of any licensed CVB3 vaccine motivated generation of an exosome-based viral vaccine, Exo-VP1. Exosomes were isolated from the culture media of VP1 expressing 293T cells stable cell lines. The prophylactic effect of Exo-VP1 was compared with a previously reported recombinant VP1 protein (rVP1) vaccine by the same research group (45). Only 2 micro-grams of the VP1 protein from Exo-VP1 vaccine triggered substantially intense virus-specific humoral and cellular immunity including cytokine release, highlighting the potency of the exosome-based CVB3 vaccine (46).

Another deadly pathogen, Zika virus, continues to be under global public health radar, as it causes severe congenital brain abnormalities in unborn fetus and neurological complications, such as, Guillain-Barré syndrome in adults (47). Since there is lack of both effective antivirals or potent vaccine against Zika virus infection due to several developmental and applicability challenges, EV-based strategy seems lucrative. Towards this end, engineered EVs expressing interferon-induced transmembrane protein 3 (IFITM3) successfully transferred the host factor across placenta and demonstrated 2-log decrease in Zika virus levels in pregnant mice blood, as well as chief organs of the developing fetus (48). This potent therapeutic approach can be adapted to design targeted viral vaccines by altering the exogenous protein moiety.

A synergistic booster effect was observed with a recombinant ALVAC (replication-deficient canarypox virus) vector based and subunit EV-based vaccine against Japanese encephalitis virus (JEV) infection. Modified viral vector expressing prM, E and NS1 genes (vCP107) of JEV was shown to generate robust anti-JEV neutralizing antibodies after receiving booster dose of the engineered EVs expressing JEV prM/M and E proteins in mice (49). Another innovative approach utilized immunomagnetic nanographene elements to engineer functional dendritic cell (DC)-extracted exosomes expressing H-2D^b^-restricted immunodominant RSV peptides (M_187–195_ and NS1_61-75_) that stimulated interferon-gamma release from RSV-primed CD8+ T cells (50). An interesting study exhibited pan-Dengue immunity by heterologous expression of envelope E domain III proteins (EDIIIconA and EDIIIconB) via engineered *Staphylococcus aureus* strain (RN4220-Δagr) membrane vesicles (MVs) in mice. Further, immunized mice sera conferred protection to Vero cells and suckling mice against lethal DENV-2 challenge. The findings demonstrate an effective strategy for producing polyvalent nanosized viral vaccines (51). Engineered exosomes carrying HPV-E7 triggered strong CD8+ T cell immunity, generating anti-HPV E7 CTLs and effectively controlling tumor growth in mouse models (52). An OVA-Texo and Gag-Texo vaccine strategy effectively targeted adenovirus-OVA and HIV, reversing CTL exhaustion, restoring immune function, and inducing potent therapeutic CD8+ T cell responses against HIV-1 infection (53). These potent vaccine strategies have potential applicability to other viral diseases of global concern.

### EV-vaccines against veterinary viruses

A novel vaccine strategy using serum EV-enriched fractions safely targeted Porcine Reproductive and Respiratory Syndrome Virus (PRRSV), eliciting specific IgG responses in swine and demonstrating promising efficacy against this economically relevant viral disease (54). Another effective strategy highlighted the role of exosomes in delivery of small RNA to pig cells and a promising anti-PRRSV vaccine strategy. Sn- and CD163-targeted amiRNAs were delivered via recombinant adenoviruses and exosomes significantly inhibited receptor expression, reduced PRRSV replication across strains, and reversed CTL dysfunction (55). M1-polarized chicken macrophage EVs proved to be effective adjuvants for live-attenuated infectious bronchitis virus (IBV) vaccine, by considerably enhancing macrophage phagocytosis, activation of cellular immunity, and cytokine release in IBV-challenged chickens (56).

### EV-vaccines against other microorganisms

Engineered EVs have also been used to enhance the immunogenicity by ensuring no degradation of oral Salmonella vaccine occurs in gut. A low-endotoxin *Salmonella choleraesuis*–derived BBV platform, functionalized for dual antigen coupling and stabilized with chitosan oligosaccharides, enabled oral delivery of subunit vaccines. It elicited robust systemic and mucosal immunity in mice, conferring complete protection against viral and bacterial pathogens, demonstrating a versatile vaccine strategy (57).

## Conclusions

EVs offer infinite potential as potent prophylactic and therapeutic agents. Their inherent biophysical attributes—size, structure, and composition—closely resemble those of resident host cells and organs, enabling natural compatibility and targeted delivery. The adaptability of EVs to diverse engineering strategies, specifically as immunogen carriers to various biological molecules, positions them as powerful vaccine delivery vehicles. This systematic summarized account of EV-based vaccine approaches against various viral diseases provides a comprehensive foundation to interested researchers and academicians, who wish to harness the benefits of EVs in vaccinology. Importantly, the strategies discussed here may be extended to other pathogens of concern or further refined to suit specific biomedical applications.

## References

[1] Welsh JA Goberdhan DCI O'Driscoll L Buzas EI Blenkiron C Bussolati B . Minimal information for studies of extracellular vesicles (MISEV2023): From basic to advanced approaches. J Extracell Vesicles. (2024) 13:e12404. doi: 10.1002/jev2.12404 38326288 PMC10850029

[2] Ibrahim SA Khan YS . Histology, extracellular vesicles. In: StatPearls. StatPearls Publishing, Treasure Island (FL (2023). 32965927

[3] Rufino-Ramos D Albuquerque PR Carmona V Perfeito R Nobre RJ Pereira de Almeida L . Extracellular vesicles: novel promising delivery systems for therapy of brain diseases. J Ctrl Release. (2017) 262:247–58. doi: 10.1016/j.jconrel.2017.07.001 28687495

[4] Corsello T Qu Y Ivanciuc T Garofalo RP Casola A . Antiviral activity of extracellular vesicles derived from respiratory syncytial virus-infected airway epithelial cells. Front Immunol. (2022) 13:886701. doi: 10.3389/fimmu.2022.886701 36032066 PMC9412241

[5] Menay F Cocozza F Gravisaco MJ Elisei A Re JI Ferella A . Extracellular vesicles derived from antigen-presenting cells pulsed with foot and mouth virus vaccine-antigens act as carriers of viral proteins and stimulate B cell response. Front Immunol. (2024) 15:1440667. doi: 10.3389/fimmu.2024.1440667 39176090 PMC11338771

[6] Alcon-LePoder S Drouet MT Roux P Frenkiel MP Arborio M Durand-Schneider AM . The secreted form of dengue virus nonstructural protein NS1 is endocytosed by hepatocytes and accumulates in late endosomes: implications for viral infectivity. J Virol. (2005) 79:11403–11. doi: 10.1128/JVI.79.17.11403-11411.2005 16103191 PMC1193635

[7] Puerta-Guardo H Glasner DR Espinosa DA Biering SB Patana M Ratnasiri K . Flavivirus NS1 triggers tissue-specific vascular endothelial dysfunction reflecting disease tropism. Cell Rep. (2019) 26:1598–1613.e8. doi: 10.1016/j.celrep.2019.01.036 30726741 PMC6934102

[8] Safadi DE Lebeau G Lagrave A Mélade J Grondin L Rosanaly S . Extracellular vesicles are conveyors of the NS1 toxin during dengue virus and Zika virus infection. Viruses. (2023) 15:364. doi: 10.3390/v15020364 36851578 PMC9965858

[9] Dutta SK Langenburg T . A perspective on current flavivirus vaccine development: A brief review. Viruses. (2023) 15:860. doi: 10.3390/v15040860 37112840 PMC10142581

[10] Fikatas A Dehairs J Noppen S Doijen J Vanderhoydonc F Meyen E . Deciphering the role of extracellular vesicles derived from ZIKV-infected hcMEC/D3 cells on the blood-brain barrier system. Viruses. (2021) 13:2363. doi: 10.3390/v13122363 34960632 PMC8708812

[11] Wang J Teng Y Zhao G Li F Hou A Sun B . Exosome-mediated delivery of inducible miR-423-5p enhances resistance of MRC-5 cells to rabies virus infection. Int J Mol Sci. (2019) 20:1537. doi: 10.3390/ijms20071537 30934732 PMC6479321

[12] Li Q Xing H Naeem A Zhang K Zheng A Huang Y . Extracellular vesicle-based mRNA therapeutics and vaccines. Explor Beijing. (2025) 5:20240109. doi: 10.1002/EXP.20240109 41476648 PMC12752554

[13] Schank MB Zhao J Wang L Moorman JP Yao ZQ . Extracellular vesicles: A comprehensive review of their origins, functions, and therapeutic potential. Biomedicines. (2026) 14:495. doi: 10.3390/biomedicines14030495 41898142 PMC13023759

[14] Lu M Xing H Zhao X Huang Y Zheng A Liang X-J . Engineered extracellular vesicles as a next-generation vaccine platform. Matter. (2024) 7:4180–205. doi: 10.1016/j.matt.2024.09.012 38826717

[15] Tan L Xing H Fang F Huang Y Lu M . Extracellular vesicle-based vaccines and immunotherapeutics for treatment of cancer. Interdiscip Med. (2026) 4:e70084. doi: 10.1002/inmd.70084 41531421

[16] Luo X McAndrews KM Arian KA Morse SJ Boeker V Kumbhar SV . Development of an engineered extracellular vesicles-based vaccine platform for combined delivery of mRNA and protein to induce functional immunity. J Ctrl Release. (2024) 374:550–62. doi: 10.1016/j.jconrel.2024.08.017 39146981 PMC11978227

[17] Ingham RJ Colwill K Howard C Dettwiler S Lim CS Yu J . WW domains provide a platform for the assembly of multiprotein networks. Mol Cell Biol. (2005) 25:7092–106. doi: 10.1128/MCB.25.16.7092-7106.2005 16055720 PMC1190255

[18] Choi S Yang Z Wang Q Qiao Z Sun M Wiggins J . Displaying and delivering viral membrane antigens via WW domain-activated extracellular vesicles. Sci Adv. (2023) 9:eade2708. doi: 10.1126/sciadv.ade2708 36706192 PMC9882979

[19] Sahin F Atasoy BT Yalcin S Bitirim VC . Membrane-targeted immunogenic compositions using exosome mimetic approach for vaccine development against SARS-CoV-2 and other pathogens. Sci Rep. (2025) 15:10899. doi: 10.1038/s41598-025-95503-y 40157987 PMC11954949

[20] ZAruba M Roschitz L Sami H Ogris M Gerner W Metzner C . Surface modification of E. coli outer membrane vesicles with glycosylphosphatidylinositol-anchored proteins: Generating pro/eukaryote chimera constructs. Membranes Bsl. (2021) 11:428. doi: 10.3390/membranes11060428 34199851 PMC8228533

[21] Valero-Rello A Sanjuán R . Enveloped viruses show increased propensity to cross-species transmission and zoonosis. Proc Natl Acad Sci USA. (2022) 119:e2215600119. doi: 10.1073/pnas.2215600119 36472956 PMC9897429

[22] Sharma J Rajput R Bhatia M Arora P Sood V . Clinical predictors of COVID-19 severity and mortality: A perspective. Front Cell Infect Microbiol. (2021) 11:674277. doi: 10.3389/fcimb.2021.674277 34760713 PMC8573222

[23] Han C Kim S Seo Y Lim M Kwon Y Yi J . Cell-engineered virus-mimetic nanovesicles for vaccination against enveloped viruses. J Extracell Vesicles. (2024) 13:e12438. doi: 10.1002/jev2.12438 38659363 PMC11043678

[24] García-Trujillo M Lavado-García J Boix-Besora A Gòdia F Cervera L . Gag HIV-1 virus-like particles and extracellular vesicles functionalization with spike epitopes of SARS-CoV-2 using a copper-free click chemistry approach. Bioconjug Chem. (2025) 36:486–99. doi: 10.1021/acs.bioconjchem.4c00559 39993141 PMC12123617

[25] Jiang L Driedonks TAP Jong WSP Dhakal S Bart van den Berg van Saparoea H Sitaras I . A bacterial extracellular vesicle-based intranasal vaccine against SARS-CoV-2 protects against disease and elicits neutralizing antibodies to wild-type and Delta variants. J Extracell Vesicles. (2022) 11:e12192. doi: 10.1002/jev2.12192 35289114 PMC8920961

[26] Jackson HK Long HM Yam-Puc JC Palmulli R Haigh TA Gerber PP . Bioengineered small extracellular vesicles deliver multiple SARS-CoV-2 antigenic fragments and drive a broad immunological response. J Extracell Vesicles. (2024) 13:e12412. doi: 10.1002/jev2.12412 38339765 PMC10858312

[27] Pomatto MAC Gai C Negro F Massari L Deregibus MC De Rosa FG . Oral delivery of mRNA vaccine by plant-derived extracellular vesicle carriers. Cells. (2023) 12:1826. doi: 10.3390/cells12141826 37508491 PMC10378442

[28] Young Chung J Thone MN Davies JE Gach JS Huw Davies D Forthal DN . Vaccination against SARS-CoV-2 using extracellular blebs derived from spike protein-expressing dendritic cells. Cell Immunol. (2023) 386:104691. doi: 10.1016/j.cellimm.2023.104691 36822152 PMC9933546

[29] Ferrantelli F Chiozzini C Manfredi F Leone P Spada M Di Virgilio A . Strong SARS-CoV-2 N-specific CD8+ T immunity induced by engineered extracellular vesicles associates with protection from lethal infection in mice. Viruses. (2022) 14:329. doi: 10.3390/v14020329 35215922 PMC8879411

[30] Manfredi F Chiozzini C Ferrantelli F Leone P Pugliese K Spada M . Antiviral effect of SARS-CoV-2 N-specific CD8+ T cells induced in lungs by engineered extracellular vesicles. NPJ Vaccines. (2023) 8:83. doi: 10.1038/s41541-023-00686-y 37268624 PMC10237059

[31] Cummings SE Delaney SP St-Denis Bissonnette F Stalker A Muradia G Mehic J . SARS-CoV-2 antigen-carrying extracellular vesicles activate T cell responses in a human immunogenicity model. iScience. (2023) 27:108708. doi: 10.1016/j.isci.2023.108708 38226155 PMC10788222

[32] Wang Z Popowski KD Zhu D de Juan Abad BL Wang X Liu M . Exosomes decorated with a recombinant SARS-CoV-2 receptor-binding domain as an inhalable COVID-19 vaccine. Nat BioMed Eng. (2022) 6:791–805. doi: 10.1038/s41551-022-00902-5 35788687 PMC10782831

[33] Wang Z Hu S Popowski KD Liu S Zhu D Mei X . Inhalation of ACE2-expressing lung exosomes provides prophylactic protection against SARS-CoV-2. Nat Commun. (2024) 15:2236. doi: 10.1038/s41467-024-45628-x 38472181 PMC10933281

[34] Cacciottolo M Li Y Nice JB LeClaire MJ Twaddle R Mora CL . Nanograms of SARS-CoV-2 spike protein delivered by exosomes induce potent neutralization of both delta and omicron variants. PloS One. (2023) 18:e0290046. doi: 10.1371/journal.pone.0290046 37607200 PMC10443850

[35] Bansal S Perincheri S Fleming T Poulson C Tiffany B Bremner RM . Cutting edge: Circulating exosomes with COVID spike protein are induced by BNT162b2 (Pfizer-BioNTech) vaccination prior to development of antibodies: A novel mechanism for immune activation by mRNA vaccines. J Immunol. (2021) 207:2405–10. doi: 10.4049/jimmunol.2100637 34654691 PMC11073804

[36] Rajput R Khanna M Kumar P Kumar B Sharma S Gupta N . Small interfering RNA targeting the nonstructural gene 1 transcript inhibits influenza A virus replication in experimental mice. Nucleic Acid Ther. (2012) 22:414–22. doi: 10.1089/nat.2012.0359 23062009

[37] Rajput R Sharma G Rawat V Gautam A Kumar B Pattnaik B . Diagnostic potential of recombinant scFv antibodies generated against hemagglutinin protein of influenza A virus. Front Immunol. (2015) 6:440. doi: 10.3389/fimmu.2015.00440 26388868 PMC4557041

[38] Lee TY Kim CU Bae EH Seo SH Jeong DG Yoon SW . Outer membrane vesicles harboring modified lipid A moiety augment the efficacy of an influenza vaccine exhibiting reduced endotoxicity in a mouse model. Vaccine. (2017) 35:586–95. doi: 10.1016/j.vaccine.2016.12.025 28024958 PMC7115551

[39] Dong C Wei L Zhu W Kim JK Wang Y Omotara P . Mature dendritic cell-derived extracellular vesicles are potent mucosal adjuvants for influenza hemagglutinin vaccines. ACS Nano. (2025) 19:25526–42. doi: 10.1021/acsnano.5c08831 40591610 PMC12269352

[40] Watkins HC Rappazzo CG Higgins JS Sun X Brock N Chau A . Safe recombinant outer membrane vesicles that display M2e elicit heterologous influenza protection. Mol Ther. (2017) 25:989–1002. doi: 10.1016/j.ymthe.2017.01.010 28215994 PMC5383554

[41] Rock KL Shen L . Cross-presentation: underlying mechanisms and role in immune surveillance. Immunol Rev. (2005) 207:166–83. doi: 10.1111/j.0105-2896.2005.00301.x 16181335

[42] Loch S Tampe´ R . Viral evasion of the MHC class I antigen processing machinery. Pflugers Arch. (2005) 451:409–17. doi: 10.1007/s00424-005-1420-8 16086162

[43] Testa JS Apcher GS Comber JD Eisenlohr LC . Exosome-driven antigen transfer for MHC class II presentation facilitated by the receptor binding activity of influenza hemagglutinin. J Immunol. (2010) 185:6608–16. doi: 10.4049/jimmunol.1001768 21048109 PMC3673890

[44] Khanna M Gautam A Rajput R Sharma L . Natural products as a paradigm for the treatment of Coxsackievirus - induced myocarditis. Curr Top Med Chem. (2020) 20:607–16. doi: 10.2174/1568026620666200129094516 31995007

[45] Gao Y Yue Y Xiong S . An albumin-binding domain peptide confers enhanced immunoprotection against viral myocarditis by CVB3 VP1 vaccine. Front Immunol. (2021) 12:666594. doi: 10.3389/fimmu.2021.666594 34630378 PMC8492941

[46] Zhang C Zhang Y Li Y Lu J Xiong S Yue Y . Exosome-based delivery of VP1 protein conferred enhanced resistance of mice to CVB3-induced viral myocarditis. Virology. (2023) 579:46–53. doi: 10.1016/j.virol.2022.12.015 36603532

[47] Rajput R Sharma J . SARS-CoV-2 in pregnancy: Fitting into the existing viral repertoire. Front Glob Womens Health. (2021) 2:647836. doi: 10.3389/fgwh.2021.647836 34816202 PMC8594046

[48] Zou X Yuan M Zhang T Zheng N Wu Z . EVs containing host restriction factor IFITM3 inhibited ZIKV infection of fetuses in pregnant mice through trans-placenta delivery. Mol Ther. (2021) 29:176–90. doi: 10.1016/j.ymthe.2020.09.026 33002418 PMC7791082

[49] Konishi E Pincus S Paoletti E Shope RE Wason PW . Avipox virus-vectored Japanese encephalitis virus vaccines: Use as vaccine candidates in combination with purified subunit immunogens. Vaccine. (1994) 12:633–8. doi: 10.1016/0264-410x(94)90269-0 8085382

[50] Hong S Ruan S Greenberg Z He M McGill JL . Development of surface engineered antigenic exosomes as vaccines for respiratory syncytial virus. Sci Rep. (2021) 11:21358. doi: 10.1038/s41598-021-00765-x 34725399 PMC8560785

[51] Yuan J Yang J Hu Z Yang Y Shang W Hu Q . Safe staphylococcal platform for the development of multivalent nanoscale vesicles against viral infections. Nano Lett. (2018) 18:725–33. doi: 10.1021/acs.nanolett.7b03893 29253342

[52] Di Bonito P Ridolfi B Columba-Cabezas S Giovannelli A Chiozzini C Manfredi F . HPV-E7 delivered by engineered exosomes elicits a protective CD8^+^ T cell-mediated immune response. Viruses. (2015) 7:1079–99. doi: 10.3390/v7031079 25760140 PMC4379561

[53] Wang R Xu A Zhang X Wu J Freywald A Xu J . Novel exosome-targeted T-cell-based vaccine counteracts T-cell anergy and converts CTL exhaustion in chronic infection via CD40L signaling through the mTORC1 pathway. Cell Mol Immunol. (2017) 14:529–45. doi: 10.1038/cmi.2016.23 27264687 PMC5518816

[54] Montaner-Tarbes S Novell E Tarancón V Borrás FE Montoya M Fraile L . Targeted-pig trial on safety and immunogenicity of serum-derived extracellular vesicles enriched fractions obtained from Porcine Respiratory and Reproductive virus infections. Sci Rep. (2018) 8:17487. doi: 10.1038/s41598-018-36141-5 30504834 PMC6269534

[55] Zhu L Song H Zhang X Xia X Sun H . Inhibition of porcine reproductive and respiratory syndrome virus infection by recombinant adenovirus- and/or exosome-delivered the artificial microRNAs targeting sialoadhesin and CD163 receptors. Virol J. (2014) 11:225. doi: 10.1186/s12985-014-0225-9 25522782 PMC4279792

[56] Zhou J Cai S Huang H Yang F Pan K Sun Z . LPS/TLR4-activated M1-polarized macrophage-derived exosomes enhance IBV vaccine efficacy in chickens. J Virol. (2025) 99:e0115625. doi: 10.1128/jvi.01156-25 40985722 PMC12548463

[57] Shen X Wang S Qiu K Liu Z Tian X Meng F . Engineered low-endotoxin bacterial biomimetic vesicles for enhanced oral dual-antigen subunit vaccine delivery. J Extracell Vesicles. (2025) 14:e70207. doi: 10.1002/jev2.70207 41316990 PMC12663866

